# Enhanced Synthesis of Antioxidant Enzymes, Defense Proteins and Leghemoglobin in Rhizobium-Free Cowpea Roots after Challenging with *Meloydogine incognita*

**DOI:** 10.3390/proteomes2040527

**Published:** 2014-11-26

**Authors:** Jose T. A. Oliveira, Jose H. Araujo-Filho, Thalles B. Grangeiro, Darcy M. F. Gondim, Jeferson Segalin, Paulo M. Pinto, Celia R. R. S. Carlini, Fredy D. A. Silva, Marina D. P. Lobo, Jose H. Costa, Ilka M. Vasconcelos

**Affiliations:** 1 Department of Biochemistry and Molecular Biology, Federal University of Ceara, Fortaleza 60451-970, Brazil; E-Mails: darcymayra@hotmail.com (D.M.F.G.); fredydavi@hotmail.com (F.D.A.S.); costajhe@yahoo.com.br (J.H.C.); imvasco@ufc.br (I.M.V.); 2 Department of Biological Sciences, State University of Rio Grande do Norte, Rio Grande do Norte, Mossoro 59610-210, Brazil; E-Mail: heliofilho10@uol.com.br; 3 Department of Biology, Federal University of Ceara, Fortaleza 60451-970, Brazil; E-Mails: thalles@ufc.br (T.B.G.); marinadplobo@gmail.com (M.D.P.L.); 4 University of Fortaleza (UNIFOR), Ceara, Fortaleza 60451-970, Brazil; 5 Department of Biophysics and Center of Biotechnology, Federal University of Rio Grande do Sul, Rio Grande do Sul, Porto Alegre 91501-970, Brazil; E-Mails: jsegalin@inmetro.rs.gov.br (J.S.); ccarlini@ufrgs.br (C.R.R.S.C.); 6 Institute of Biotechnology, University of Caxias do Sul, Caxias do Sul, RS, Rio Grande do Sul, Caxias do Sul 95070-560, Brazil; E-Mail: paulomarcospinto@gmail.com

**Keywords:** cowpea, defense proteins, *Meloidogyne incognita*, plant proteomics

## Abstract

The root knot nematodes (RKN), *Meloydogine* spp., particularly *Meloidogyne incognita* and *Meloidogyne javanica* species, parasitize several plant species and are responsible for large annual yield losses all over the world. Only a few available chemical nematicides are still authorized for RKN control owing to environmental and health reasons. Thus, plant resistance is currently considered the method of choice for controlling RKN, and research performed on the molecular interactions between plants and nematodes to identify genes of interest is of paramount importance. The present work aimed to identify the differential accumulation of root proteins of a resistant cowpea genotype (CE-31) inoculated with *M. incognita* (Race 3) in comparison with mock-inoculated control, using 2D electrophoresis assay, mass spectrometry identification and gene expression analyses by RT-PCR. The results showed that at least 22 proteins were differentially represented in response to RKN challenge of cowpea roots mainly within 4–6 days after inoculation. Amongst the up-represented proteins were SOD, APX, PR-1, β-1,3-glucanase, chitinases, cysteine protease, secondary metabolism enzymes, key enzymes involved in ethylene biosynthesis, proteins involved in MAPK pathway signaling and, surprisingly, leghemoglobin in non-rhizobium-bacterized cowpea. These findings show that an important rearrangement in the resistant cowpea root proteome occurred following challenge with *M. incognita*.

## 1. Introduction

The root knot nematodes (RKN, *Meloidogyne* spp.) are among the most damaging plant parasites, as they establish feeding sites in the roots of major crops, preventing the normal uptake of water and nutrients. They are responsible for large annual yield losses all over the world [1], and their economic importance is increasing, as only a few available chemical nematicides are still authorized for RKN control, owing to environmental and health reasons. Thus, plant resistance is currently considered the method of choice for controlling root-knot nematodes, and research performed on the molecular interactions between plants and nematodes to identify genes of interest is of paramount importance.

RKNs are obligate biotrophic pathogens that establish and maintain permanent feeding cells, the giant cells, inside the root system of host plants, from which they draw off nutrients to complete their life cycle. The giant cells result from repeated rounds of karyokinesis without cell division. Hyperplasia and hypertrophy of the cells surrounding the feeding sites lead to the formation of tumors designated as root galls, the primary visible symptom of infection [1]. These symptoms occur in susceptible plants, presumably because they do not perceive the enemy nor activate their defense mechanisms efficiently. Resistant plants can trigger plant immune responses, as they possess the pattern recognition receptors (PRR) that recognize conserved pathogen-derived molecules, the pathogen- or microbe-associated molecular patterns (PAMPs/MAMPs) and/or possess R proteins (NB-LRR proteins) composed of a central nucleotide-binding site (NBS) and a C-terminal leucine-rich repeat (LRR), which detect pathogen effectors [2]. Most of the several plant proteins conferring resistance to nematodes have been identified as NB-LRR proteins [3]. After recognition, transcriptional reprogramming of the plant is induced by the nematode, both locally and systemically throughout the plant [4]. In various incompatible relationships between pathogens and the resistant plant, one of the first events observed after recognition is the oxidative burst, during which a rapid generation of reactive oxygen species (ROS), such as superoxide anion (O_2_^−^), hydrogen peroxide (H_2_O_2_) and also nitric oxide (NO), occur locally in the site of attempted infection. ROS generation is often associated with the hypersensitive response (HR), a programmed cell death (PCD) process that occurs around the infection site [5] as a plant attempts to hamper the pathogen invasion. An excess of ROS generated during HR causes considerable cell damage, but plants can activate various mechanisms for the efficient scavenging of these transient augmentations in ROS. These include the non-enzymatic antioxidant systems, such as ascorbate and glutathione, and the enzymatic ROS-scavenging mechanisms in which catalase, peroxidase, ascorbate peroxidase, superoxide dismutase, glutathione peroxidases and peroxiredoxins participate. Transiently elevated ROS levels have also been considered as second messengers in plant, as they are perceived by different receptors, proteins or enzymes and seem to be involved with the regulation of phytohormones, such as ethylene (ET), salicylic acid (SA) and jasmonic acid (JA), which play important roles in plant-pathogen interactions [6].

After HR, a second kind of induced response against pathogen attack, the systemic acquired response (SAR), takes place, in which various defense genes are over- or down-regulated, mainly by intervention of SA, JA and ET [6]. Das *et al.* [7] showed that 552 genes were significantly differentially expressed between the *M. incognita*-infected and non-infected resistant cowpea CB46 plants and amongst the upregulated genes, there were those involved in metabolism (42.8%), genes coding for proteins with binding functions (25.3%) and genes involved in the interaction with the environment (15.8%), whereas those gene downregulated the code for proteins with binding functions (34.7%), metabolism (29.6%) and protein fate (20.3%).

The cowpea (*Vigna unguiculata* (L.) Walp.) legume is an important crop used as food mostly in tropical and semi-arid regions of the world. The dried seeds, leaves, immature seeds and fresh green pods are all consumed. However, the cowpea seeds represent the major form of utilization, because of their nutritional profile, particularly protein (20.3%–29.3%) and carbohydrate (55.6%–74.5%) contents [8]. The resistance of cowpeas to *M. incognita* resides on a single gene or locus, designated Rk, with alleles rk, rki, Rk, Rk2 and Rk3, which effectively inhibit the reproduction of *M. incognita* [9]. The cowpea genotype CE-31 is highly resistant to *Meloydogine incognita* Race 3 [10].

The aim of this work was to analyze the differential accumulation of proteins in the roots of the resistant cowpea genotype CE-31 inoculated with *M. incognita* (Race 3) and non-inoculated control, using a 2D electrophoresis assay associated with mass spectrometry identification and gene expression analyses by reverse transcription-polymerase chain reaction (RT-PCR).

## 2. Experimental Section

### 2.1. Nematode Inoculums

The root knot nematode (RKN) inoculum was obtained from a population of *Meloidogyne incognita* (Race 3) isolated from susceptible cowpea plants (cv. Vita-3), growing in 1.5-L plastic pots containing exhaustive tap water washed river bottom sand that was previously mixed with humus (5:1, m/m) and autoclaved (121 °C, 30 min, 1.5 kgf/cm^2^ (a kilogram-force per square centimeter)). Plants were maintained in a greenhouse, where the average temperature varied from 25 °C (night) to 35 °C (day), relative humidity (RH) from 55% (day) to 80% (night) and natural light *ca*. 700 µmol·m^−2^·s^−1^ of photosynthetically active radiation (PAR) at the plant canopy. Irrigation was done daily with distilled water for up to 4 days after sowing, followed by irrigation (100 mL/pot) with 5-times diluted nutritive Hoagland and Arnon solution, as previously described [11]. Egg masses from *M. incognita* were isolated from galled cowpea roots using a stylet under a stereoscopic microscope (ausJENA, Jena, Germany). The egg masses were sterilized by immersion in sodium hypochlorite (0.05%, v/v active chloride) for 3 min, followed by three washings with sterile Milli-Q grade water [12]. Next, they were placed in sterile Milli-Q grade water in a Petri dish, and the infective, motile second-stage juveniles (J2) were allowed to hatch at around 26 °C in the dark. The J2 hatched within 24 h were discarded, and those of 48 and 72 h were collected every 12 h, concentrated using a 30-µm pore size nylon sieve and resuspended in sterile Milli-Q grade water to a 2000 J2/mL population that was used as the inoculum within 1 to 3 days of collection [13].

### 2.2. Plant Material and Nematode Inoculation

The cowpea seeds from cv. CE-31, highly resistant to *M. incognita* (Race 3) [10], were surface sterilized with sodium hypochlorite (0.05% active chloride) for 5 min, washed exhaustively with sterile Milli-Q grade water and germinated between two moist filter papers (Germitest^TM^), which were placed in a plastic tray in the dark. The filter papers were watered with sterile Milli-Q grade water twice a day. Three days later, the seeds that had germinated were selected and transplanted to 1.5-L plastic jars (five per jar) containing river bottom sand thoroughly washed with tap water and autoclaved (121 °C, 30 min, 1.5 kgf/cm^2^). The jars were kept inside a four-legged aluminum framework covered with an air permeable and transparent nylon net to protect the seedlings from dust and insects and maintained in a greenhouse under the same conditions of irrigation, temperature, photoperiod and PAR, as described above. Twelve days after seedling transplantation to the jars (15 days after planting), two plants were removed from each jar, and the three remaining ones inoculated with 2000 *M. incognita* J2 suspended in 2 mL of sterile water. The J2 suspension was placed in a 2-cm deep hole in the soil neighboring the main root axis of each plant and the hole filled with river sand. Controls were mock-inoculated with sterile water. The jars were arranged in a completely randomized-block design experiment, with twelve plants and three repetitions for each studied time point. Twelve plants were harvested at each time point (0, 12, and 24 h and 2, 4, 6, 8 and 10 days after inoculation). The plants were uprooted and the roots washed free of soil with distilled water, dried between two layers of paper towel, frozen in liquid nitrogen, powdered and stored at −80 °C for posterior use.

### 2.3. Extraction of Proteins from the Cowpea Roots

The frozen root powder (4.0 g) was resuspended in 8.0 mL of 100 mM Tris-HCl buffer, pH 8.0, containing 20% (v/v) glycerol, 3% (v/v) PEG, polyvinylpolypyrrolidone (PVPP) (1:2, m/v), 10.0 mM EDTA, 1.0 mM DTT (Dithiothreitol) and 1.0 mM PMSF (phenylmethylsulfonyl fluoride). After 2 h under gentle agitation at 4 °C, the suspension was centrifuged at 20,000× *g* for 20 min, 4 °C, the supernatant collected and the extracted proteins precipitated overnight at −80 °C with 30% (v/v) TCA (Trichloroacetic acid) in acetone. The proteins precipitated were centrifuged at 5000× *g* for 10 min, 4 °C, and the pellet washed twice in methanol, twice in acetone, vacuum dried and resuspended in 7 M urea/2 M thiourea. The final homogenate was centrifuged as above, the supernatant collected and the protein concentration measured using BSA as the standard [14]. To detect any possible contamination of the RKN-inoculated root protein samples with proteins originating from the nematode itself, the same above procedure to extract and process the root proteins was used to extract the nematode proteins from a mixture of 2000 J2 + 50 females + 50 eggs masses, and 2D electrophoresis gels were produced. The amount of J2, female and egg masses tested was about 50-times higher than those found, on average, in the roots of the resistant genotype, CE-31, infected with the *M. incognita* nematode [10].

### 2.4. Two-Dimensional Polyacrylamide Gel Electrophoresis (2D-SDS-PAGE)

Root proteins (200 µg) in 250 µL of 7 M urea, 2 M thiourea, 0.065 M DTT, 0.5% (m/v) CHAPS (3-[(3-cholamidopropyl)dimethylammonio]-1-propanesulfonate), 0.5% (m/v) IPG buffer and 0.002% (m/v) bromophenol blue were loaded onto 13-cm IPG immobilized pH (4–7) gradient strips (GE Healthcare, Uppsala, Sweden). The strips were rehydrated for 10 h (overnight) at 25 °C and isoelectric focused in an Ettan IPGphor II system (Amersham Biosciences, Piscataway, NJ, USA) programmed as follows: 200 V for 1 h, 500 V for 2 h, 5000 V for 2.5 h and, finally, 10,000 V for 1 h to achieve a total of 31.4 kVh. After isoelectric focusing, the strips were equilibrated for 15 min by shaking in 1.5 M Tris-HCL buffer, pH 8.8, containing 6 M urea, 30% (v/v) glycerol, 2% (m/v) SDS, 2% (m/v) DTT and trace amounts of bromophenol blue. These same strips were re-equilibrated for a further 15 min in the above buffer, except that it contained 2.5% (m/v) iodoacetamide instead of DTT. For 2D-electrophoresis [15], the strip was fitted on a 15% (m/v) acrylamide gel (150 mm × 180 mm × 1.5 mm) sealed with 0.5% (w/v) agarose prepared in the electrode buffer. Electrophoresis was carried out at 40 mA/gel, in a vertical electrophoresis SE 600 unit (18 × 16 cm, GE-Healthcare; Amersham Bioscience) coupled to a circulating bath (MultTemp II, Pharmacia, LKB, Uppsala, Sweden) set at 5 °C. Protein spots were detected by colloidal Coomassie blue stain [16] and scanned at 300 dpi (ImageScanner Amersham Bioscience). Images (.tiff) were analyzed by the ImageMaster 2-D Platinum version 6.0 software, (GE-Healthcare; Amersham Bioscience). Three 2D patterns of proteins from RKN-inoculated and mock-inoculated control were produced for each time point after inoculation and compared in order to identify common, distinct, as well as differentially represented proteins. Only the protein spots that were at least two-fold up- or down-regulated after RKN inoculation, compared with the corresponding control (non-inoculated), were excised from 2D gels for protein identification after mass spectrometry.

### 2.5. In-Gel Digestion

Upregulated protein spots were manually excised from 2D gels run with RKN-inoculated cowpea root samples, whereas those downregulated were excised from control (non-inoculated) gels where they were more prominent. Each protein spot was individually transferred to 0.6 mL tubes, fragmented to about 1 mm^3^ pieces, washed twice with ultrapure water and destained three-times with a 1:1 mixture of 25 mM ammonium bicarbonate and 50% (v/v) acetonitrile (ACN), pH 8.0, followed by two dehydration steps with 100% ACN for 5 min each. After being dried under vacuum, the fragments were rehydrated with 20 µL of the digestion solution consisting of 25 mM ammonium bicarbonate, 1 mM CaCl_2_ and 0.2 µg sequencing-grade modified trypsin (Promega, Madison, WI, USA). The reaction was done in a water bath at 37 °C for 16 h [17]. The resulting tryptic fragments were recovered by diffusion into a solution composed of 50% (v/v) ACN and 5% (v/v) trifluoroacetic acid (TFA) in 50 mM ammonium bicarbonate for 30 min, in three washes, transferred to micro tubes and dried under vacuum.

### 2.6. Mass Spectrometry Analysis

Prior to analysis, the dried peptides were dissolved in 10 μL of 0.1% formic acid. MS/MS analyses were performed by an electrospray ionization (ESI) quadrupole time-of-flight (Q-TOF) Micro™ mass spectrometer coupled to a nanoACQUITY^®^ UltraPerformance liquid chromatography system (Waters, Milford, TX, USA). The peptides were loaded on a nanoeasy-C18 (75 µm ID) capillary column equilibrated with 98% Solution A (0.1% formic acid/water) and 2% B (ACN/0.1% formic acid). Elution was done with the gradient schedule: 2%–60% B for 13 min; 60%–95% B for 6 min; 95%–2% B for 11 min. Data were acquired in data-dependent mode (DDA), and multiple charged peptide ions (+2 and +3) were automatically mass selected and dissociated in MS/MS experiments. The ionization conditions and liquid chromatography were: 0.6 µL/min flow; 3.5 kV nanoflow capillary voltage; 100 °C block temperature; 50 V as the cone voltage. The mass spectra were acquired and processed using the Mascot Distiller software (Matrix Science, London, UK), and the Mascot Generic Format (MGF) files generated were searched against the non-redundant protein sequence databases from NCBI (National Center for Biotechnology Information), using the MASCOT v. 2.2 software (Matrix Science, London, UK, www.matrixscience.com). Searches were performed using the following criteria: Viridiplantae as the taxonomic category, tolerance of one missed cleavage; cysteine carbamidomethylation; methionine oxidation; and 0.2 Da for peptide mass tolerance. The limit of significance was fixed at *p* < 0.05 and identification required that each protein contained at least one peptide with an expected value <0.05. The statistical test (ANOVA) was performed automatically. The proteins were categorized based on Bevan *et al.* [18].

### 2.7. Gene Expression Analysis

Gene expression analysis was accomplished by reverse transcription of mRNA templates coupled to *in vitro* amplification by polymerase chain reaction (RT-PCR). To this end, total RNA was extracted from RKN-inoculated and non-inoculated cowpea fresh roots at 4, 5 and 6 days after inoculation (DAI) by the Tris-lithium chloride procedure adapted from Chang *et al.* [19]. Two grams of fresh roots were ground with liquid nitrogen in a mortar and pestle and the fine powder transferred to Falcon tubes (15 mL) to which 6 mL of the extraction buffer (100 mM Tris-HCl pH 8.0 buffer, containing 2% CTAB (m/v), 2 M NaCl and 25 mM EDTA) were slowly added and gently mixed by inversion. The suspension was incubated at 25 °C for 1 h under gentle inversion and, next, an equal volume of chloroform/isoamyl alcohol solution (24:1, v/v) was added. The mixture was incubated at 25 °C for 20 min under gentle agitation and centrifuged (6500× *g*, 10 min, 25 °C). The upper phase was transferred to a new Falcon tube, to which 7.0 mL of chloroform/isoamyl alcohol were added, mixed for 5 min and centrifuged under the above conditions. The supernatant was transferred to a new Falcon tube, and lithium chloride (10 M, 1/3 of total volume) was immediately added. The nucleic acids were left to precipitate overnight at 4 °C. Next, the suspension was centrifuged (8000× *g*, 45 min, 4 °C), the supernatant discarded, the precipitate washed two times with 70% ethanol and collected by centrifugation (8000× *g*, 10 min, 4 °C). The final pellet was left to dry at 25 °C and resuspended with 250 µL of diethyl pyrocarbonate (DEPC)-treated water. The integrity of the RNA samples was checked by 1% (m/v) agarose gel electrophoresis, and the yield was estimated by measuring the absorbance at 260 nm [20].

Prior to cDNA synthesis, residual DNA was removed with RQ1 RNAase-free DNAse I (Promega) and the RNA purified with the RNeasy mini Kit (Qiagen, Hilden, Germany), according to the manufacturer’s instructions. First strand cDNA was synthesized from the mRNA present in the purified total RNA using oligo(dT)_18_ (Fermentas Life Sciences, Burlington, ON, Canada) and the ImProm-II™ Reverse Transcriptase (Promega), according to the supplier’s recommendations. The first-strand cDNA products were then amplified by PCR using oligonucleotide primers (Table 1) targeting some genes whose products showed a decreased or increased amount in RKN-inoculated roots in comparison to roots of non-inoculated plants, as detected by 2D-PAGE. Moreover, although chitinases were not identified among the selected protein spots that showed differential response in RKN-inoculated cowpea roots, primers targeting genes encoding chitinases were also designed and included in the gene expression analysis, owing to the well-known role of these proteins in plant defense. In addition, specific oligonucleotide primers targeting conserved regions of *nodC* [21], one of the genes responsible for the synthesis of the Nod-factors core structure and present in all nodulating rhizobia, were also used. This aimed at ensuring that root samples used in this study were not infected by *Rhizobium* spp. Cowpea genomic DNA was isolated by a CTAB-based protocol [22] and used as a template in pilot PCR amplifications to select the optimal annealing temperature for each pair of primers.

The reactions were performed in a final volume of 10 μL containing first-strand cDNA (750 ng), 1× GoTaq reaction buffer (Promega), 1.5 mM MgCl_2_, 200 μM of each dNTP, 0.5 μM of each primer and 1.25 U of GoTaq DNA Polymerase (Promega). Amplifications were performed in a PTC-200 thermocycler (MJ Research, Waltham, MA, USA) using the following cycling parameters: an initial denaturation step (95 °C for 2 min) followed by cycles of denaturation (95 °C, 1 min), annealing (1 min) and extension (72 °C, 3 min). The number of cycles and the annealing temperatures varied according to the target transcript (Table 1). After the last cycle, the reactions were further incubated for 10 min at 72 °C. The PCR products were analyzed by 1% (m/v) agarose gel electrophoresis, and the DNA bands were stained with 0.5 μg/mL ethidium bromide and visualized under UV light.

## 3. Results

### 3.1. Proteomic Analysis of RKN-Inoculated and Non-Inoculated Cowpea Roots

Eleven solutions/buffers for the extraction of cowpea root proteins (Table 2) were individually mixed with the cowpea genotype CE-31 root powder (1:2, m/v) to test which one better extracted the proteins for proteome analysis. Dependent on the buffer system used, the protein concentration varied from 0.11 to 0.38 mg/mL with significant differences between some of the buffers tested. As in preliminary tests, we have carried out 2D electrophoresis runs loading 100, 150, 200, 250, 300, 350 and 400 µg root protein/gel in order to choose which buffer and concentration gave the best resolution of the protein spots. Repeated protein extraction procedures for every buffer were done, and the soluble proteins were obtained, concentrated by precipitation with TCA in acetone, as described in Section 2.3, and recovered to reach the desired amounts. Buffer 9 (Table 2) extracted the highest quantity of proteins, and the best concentration to load the 2D-gels here discussed was 200 µg protein, as below this value, most of the spots were not visible; at higher protein concentrations, the gels were overloaded and lost resolution. Moreover, the Buffer 9 system apparently removes unwanted compounds, as it produced stained gels with low backgrounds that did not interfere with image acquisition (ImageScanner Amersham Bioscience), as described in Section 2.4.

**Table 1:** Nucleotide sequences of the primers ^1^ used in RT-PCR.

Target Gene (GenBank Accession Number)	Oligonucleotide Sequences ^2^	Position ^3^	Amplicon Size (bp)	Annealing Temperature (°C)/Cycle	Reference
Asparaginyl endopeptidase (D89971)	5'-AACGGCTATTGGAACTAC-3' (f)	217–234	876	52.5/35	This work
	5'-GAGATCAGCATCCCTTTG-3' (r)	1074–1092			
ACC synthase (Z12135)	5'-CAAATGGGTCTTGCTGAGAAT-3' (f)	70–90	858	58.9/20	This work
	5'-TCTCAGCCTCTCCCTGTT-3' (r)	910–927 ^4^			
ARG 10 (AB012110)	5'-CGAACACCATCGCCAAAG-3' (f)	104–121	498	56.4/35	This work
	5'-AGGGAAAGAAGCAAGCGA-3' (r)	584–601			
Chalcone-flavanone isomerase (AB073787)	5'-GAGAGGGGTTGACGATT-3' (f)	112–129	477	54.2/35	This work
	5'-GCCAATCATCGTCTCCAA-3' (r)	571–588			
Cysteinyl endopeptidase (U49445)	5'-TACGAGAGATGGAGGAGT-3' (f)	119–136	765	51.2/25	This work
	5'-TCCGACAATTGCTACACC-3' (r)	866–883			
Leghemoglobin (U33205)	5'-ATGGTTGCTTTCTCTGACAAG-3' (f)	46–66	375	64.7/35	This work
	5'-TTCATCACTCCATTTGTCTCC-3' (r)	400–420			
CuZn-superoxide dismutase (AJ278668)	5'-AAAGCGGTGGCGGTGCTGAAA-3' (f)	195–215	393	61.0/35	This work
	5'-GCTCAGTTCATGGCCGCCTTT-3' (r)	567–587			
nodC (AE006469)	5'-TGATYGAYATGGARTAYTGGCT-3' (f)	545–566	640	55.6/35	Sarita *et al.* 2005 [21]
	5'-CGYGACARCCARTCGCTRTTG-3' (r)	1164–1184			
Chitinase I (X88800)	5'-AGGATGATATGGAGCGTAGC-3' (f)	14–33	972	58.0/28	This work
	5'-GACAGGGTGAGATGTAGATC-3' (r)	966–985			
Chitinase IIIa (X88802)	5'-CTATCAACAACTGCAACGTG-3' (f)	152–171	576	55.0/28	This work
	5'-ATTTGGAAGAACCCTTGATG-3' (r)	708–727			
Chitinase IIIb (X88801)	5'-ACGTCAACATAGCTTTCCTC-3' (f)	175–194	563	55.0/28	This work
	5'-CTTCCCAGCAGGTACTGTAC-3' (r)	718–737			
Chitinase IV (X88803)	5'-GCTCAGAACTGTGGTTGTGC-3' (f)	8–27	743	57.0/28	This work
	5'-TAGCAAGTAAGATTATCACC-3' (r)	731–750			
Actin (AF143208)	5'-GCGTGATCTCACTGATGC-3' (f)	669–686	530	59.0/35	Costa *et al.*, 2004 [23]
	5'-TCGCAATCCACATCTGTTGG-3' (r)	1179–1198			

^1^ The properties of each primer, including melting temperature, percent G + C content and PCR suitability, were determined using the PCR Primer Stats tool of the Sequence Manipulation Suite version 2 program (http://www.bioinformatics.org/sms2/index.html). ^2^ The forward and reverse primers are indicated by f and r, respectively (shown in parenthesis after each primer sequence). ^3^ Primer-binding sites in the corresponding target sequences deposited in the GenBank nucleotide database. ^4^ The numbers refer to the coding sequence extracted from Z12135.

**Table 2:** Solutions and buffers used to extract the root proteins * from cowpea genotype CE-31 for two-dimensional polyacrylamide gel electrophoresis (2D-SDS-PAGE) analysis.

(1) 50 mM pyridine + 10 mM thiourea + 1% (m/v) SDS + 100 M HCl, pH 5.0
(2) 50 mM pyridine + 10 M thiourea + 1% (m/v) SDS + 100 M HCl, pH 5.0, + 1:2 (m/v) polyvinylpolypyrrolidone (PVPP)
(3) 20 mM Tris-HCl pH 6.0, containing 20% (v/v) glycerol + 3% (v/v) *polyethylene glycol* (*PEG*)
(4) 20 mM Tris-HCl pH 6.0, containing 20% (v/v) glycerol, 3% (v/v) PEG, 1:2 (m/v) PVPP
(5) 40 mM Tris-HCl pH 7.0, containing 250 mM sucrose, 1% (v/v) triton X-100, 10 mM ethylenediaminetetraacetic acid (*EDTA*), 1.0 mM DTT, 1.0 mM phenylmethylsulfonyl fluoride (PMSF)
(6) 40 mM Tris-HCl pH 7.0, containing 250 mM sucrose, 1% (v/v) Triton X-100, 10 mM EDTA, 1.0 mM dithiothreitol (DTT), 1.0 mM PMSF, 1:2 (m/v) PVPP
(7) 100 mM Tris-HCl pH 8.0, containing 20% (v/v) glycerol, 3% (v/v) PEG
(8) 100 mM Tris-HCl pH 8.0, containing 20% (v/v) glycerol, 3% (v/v) PEG, 1:2 (m/v) PVPP.
(9) 100 mM Tris-HCl pH 8.0, containing 20% (v/v) glycerol, 3% (v/v) PEG, 1:2 (m/v) PVPP, 10 mM EDTA, 1.0 mM DTT, 1.0 mM PMSF
(10) 100 mM Tris-HCl pH 9.0, containing 0.01 M EDTA, 1% (v/v) Triton X-100
(11) 100 mM Tris-HCl pH 9.0, containing 0.01 M EDTA, 1% (v/v) Triton X-100, 1:2 (m/v) PVPP

* The proportion of cowpea genotype CE-31 root powder to solutions/buffers was 1:2 (m/v).

**Figure 1:**
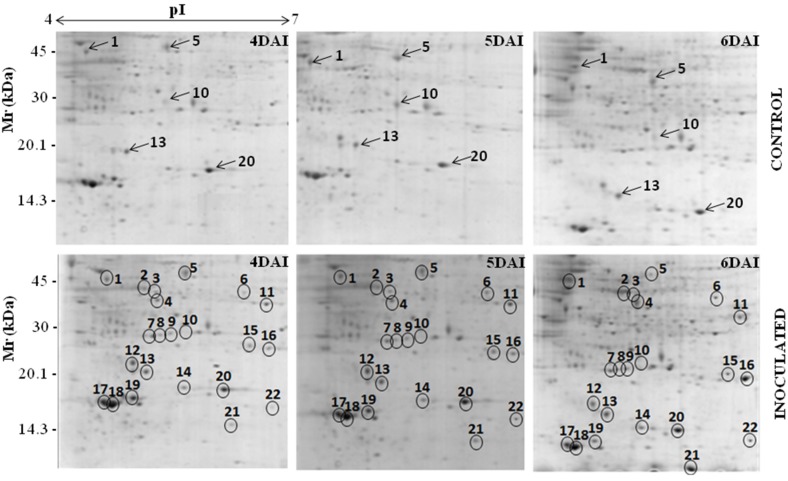
Protein profiles for control and *M. incognita*-infected cowpea genotype CE-31 roots at 4, 5 and 6 days after inoculation (DAI). Root proteins (200 μg) were extracted and separated in the first dimension by isoelectric focusing (pI 4–7) and in the second dimension by SDS-PAGE. Protein spots were detected by colloidal Coomassie blue stain [16]. Proteins that had differential accumulation in *M. incognita*-infected plants 4, 5 and 6 DAI in relation to the respective controls (mock-inoculated) are circled. Arrows in the gels of control plants indicate proteins that were down-represented after challenging with *M. incognita*.

**Figure 2:**
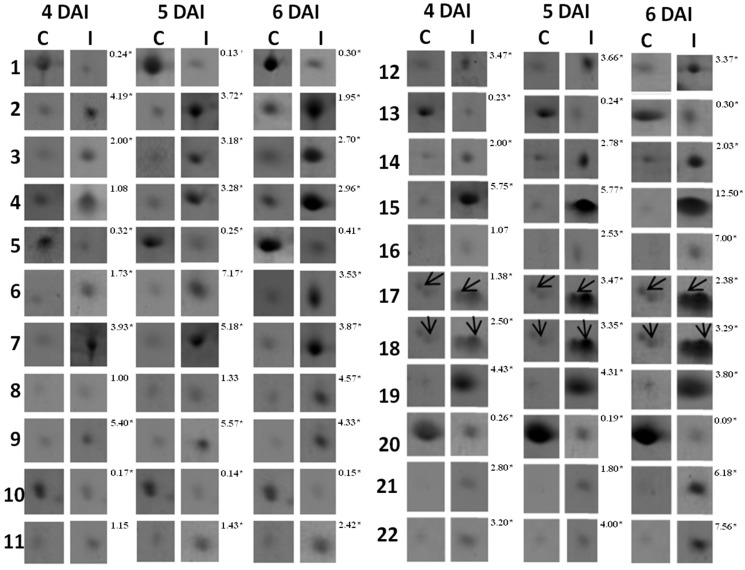
Enlarged views of the up- and down-represented proteins that were identified in the roots of the cowpea genotype CE-31 inoculated with *M. incognita* (Race 3) and the non-inoculated control. Numbers at the left correspond to those protein spots denoted in Figure 1. Arrows are placed on Spots 17 and 18 to indicate that they appear in pairs, whose levels changed in response to *M. incognita* infection. Numbers at the right of every double column denote the mean of the protein fold change measured as the difference in intensity for each spot between root knot nematode (RKN)-infected and control plants from three gels using different biological samples. The asterisk on the numbers denotes significant difference (*p* ≤ 0.05) after application of the *t*-test.

The 2D-gels obtained from three independent experiments for cowpea root samples collected at 0, 12, 24, 48 h after inoculation (HAI) and 4, 6, 8 and 10 days after inoculation (DAI) were matched. Only the spots present in all gels developed for each time point within the pI range of 4–7 were considered (Figure 1). In the RKN-infected cowpea roots compared with mock-inoculated plants (control), the protein spot alterations were more prominent between 4 and 6 DAI. About 339, 370 and 368 protein spots were detected in the control plants at 4, 5 and 6 DAI, respectively, whereas for the RKN-inoculated plants, they were around 347, 370 and 368. Taking into consideration only the protein spots that were two-fold or more altered (*p* ≤ 0.05), a total of 32 proteins were up- (26 spots) or down-represented (six spots) in the RKN-inoculated compared with the non-inoculated roots of the resistant cowpea cv. CE-31. Out of these 32 proteins spots, 22 (17 up- and five down-represented) (Figure 1 and Figure 2) were excised and further analyzed by mass spectrometry. The remaining 10 protein spots (nine up- and one down-represented), although significantly (*p <* 0.05) reprogrammed in the RKN-inoculated roots in comparison with control plants, were less abundant proteins only visualized at a certain zoom level by the ImageMaster 2-D Platinum version 6.0 software and, thus, were not excised due to technical difficulties, nor analyzed by mass spectrometry for identification. To exclude the possibility of misinterpretation because of the possible presence of RKN-derived proteins together with those originating from RKN-infected cowpea roots, 2D gels were also run only with the nematode-derived proteins under the same conditions of cowpea root sample and revealed using colloidal Coomassie or the silver nitrate staining method. However, no protein spot could be visualized, making it evident that all protein spots found in 2D gels were exclusively derived from the cowpea roots. The 22 root proteins excised from the gels were trypsinized and subjected to mass spectrometry analysis and their sequences compared for similar protein sequences deposited in the NCBI database. However, only 17 out of 22 spots were identified. Table 3 shows the identification of these proteins grouped according to their biological functions, as well as pI, molecular weight, fragment amino acid sequence, statistical scores and the percentages of coverage of their sequences. Fifteen (two downregulated and 13 upregulated) similar sequences were found. The upregulated proteins were: aminocyclopropane-1-carboxylic acid synthase (ACC synthase, Spot 2), 1-aminocyclopropane-1-carboxylate oxidase (ACC oxidase, Spot 3) cysteinyl endopeptidase (Spot 4), chalcone-flavone isomerase (Spot 6), ascorbate peroxidase (Spot 11), auxin-induced protein (Spot 12), superoxide dismutase CuZn-dependent (Spot 15), Class I heat shock protein (Spot 16), PR-1 (Spot 17), PR-3 (Spot 18), PR-2 (Spot 19), leghemoglobin (Spot 21) and nucleoside diphosphate kinase (Spot 22). The two downregulated proteins were identified as asparaginyl endopeptidase (Spot 1) and ARG 10 (Spot 10). All of the identified proteins showed score values significantly above the minimum threshold of reliability calculated automatically by the Mascot program for mass spectra analysis. These identified proteins showed similar mass spectra with those of plant species within the Fabaceae family to which *Vigna unguiculata* (cowpea) belongs. Six of them (Spots 1, 2, 3, 4, 10 and 12) showed peptide fragments similar to those of two other plant species belonging to the *Vigna* genus (*V. mungo*, *V. radiata*), and the remaining three proteins (Spots 11, 18, and 21) matched with peptides fragments of *V. unguiculata*. These findings show that the data of our study are consistent. Two protein spots (7 and 9) were matched with the protein sequences deduced *in silico*, but of unknown biological functions. Spots 5, 8, 13, 14 and 20 were not successfully identified.

**Table 3:** Identification of up- and don-represented proteins of the roots of cowpea genotype CE-31 after being challenged with *M. incognita* (Race 3) in relation to non-infected control plants.

Spot *No.	Accession No. (NCBI)	Protein Identification	Organism	pI/MW (kDa)		Score	Sequence Covered (%)	Sequences
				Experimental	Theoretical			
**Functional category: Disease/defense**								
**2↑**	gi/297493	ACC synthase	*Vigna radiata*	4.89/39.678	5.40/43.768	79	7.98	YFDGWK
								VHIVYSLSK
								VGTIYSYNDSVVTTAR
**3↑**	gi/86197901	ACC oxidase	*Vigna radiate*	5.05/38.056	5.86/40.236	111	12.30	GAAMEMIK
								EMVANK
								VSNYPPCPTPDLIK
								DDQWIDVPPMR
**11↑**	gi/42795352	Ascorbate peroxidase	*Vigna unguiculata*	6.62/28238	6.67/31.746	108	11.84	NCAPLMLR
								EIVALSGGHTLGR
								SGFDGPWTEDPLK
**15↑**	gi/13274148	CuZn-superoxide dismutase	*Populus tremula*	6.76/23.114	5.87/29.203	142	14.74	AVAVLK
								LTHGAPEDEIR
								GGHELSSTTGNAGGR
**16↑**	gi/123539	17.5 kDa HSP	*Glycine max*	5.87/22.268	6.12/17.412	177	18.18	DFHVPTSSVSAENSAFVSTR
								VLQISGER
**17↑**	gi/130829	PR-1	*Phaseolus vulgaris*	4.44/16.126	4.83/16.528	168	18.58	ALPDSFK
								ISFVEDGETK
								LSDGPNGGSLIK
**18↑**	gi/4850337	PR-3 (Chitinase)	*Vigna unguiculata*	4.55/15.971	4.75/16.265	122	14.28	ISFLEDGETK
								LSDGSNGGSVVK
**19↑**	gi/ 130835	PR-2 (β-1,3-glucanase)	*Phaseolus vulgaris*	5.11/16.352	4.85/16.402	86	10.96	ISIDSK
								GDAPPNEDELK
**Functional category: Secondary metabolism**								
**6↑**	gi/27530706	Chalcone-flavone isomerase	*Lotus japonicus*	6.41/35666	5.94 /36.987	97	11.11	SYFLGGAGER
								STGTYGEAEAAAIGK
**Functional category: Metabolism**								
**21↑**	gi/ 20138591	Leghemoglobin	*Vigna unguiculata*	6.00/14.722	5.68/15.341	225	31.03	ADIPK
								NLFSFLANGVDATNPK
								ASGGVVADAALGAVHSQK
								EAVGDK
**22↑**	gi/ 26245403	Nucleoside diphosphate Kinase	*Glycine max*	6.89/15.964	6.30/16.254	183	21.08	PDGVQSGLIGEIISR
								IIGATNPAQSEPGTIR
**Functional category: Protein destination and storage**								
**1↓**	gi/ 4589396	Asparaginyl endopeptidase	*Vigna mungo*	4.38/49.316	5.14/52.982	77	6.83	FPIIFVVANLITLVSGGR
								NSLVPPSK
								APLGSSR
**4↑**	gi/ 1223922	Cysteinyl endopeptidase	*Vigna radiata*	5.10/35.289	5.44/37.332	64	6.35	LLWVVLSLSLVLGVANSFDFHEK
**Functional category: Unknown/Predicted/Uncharacterized**								
**7↑**	gi/297849580	Predicted protein	*Arabidopsis lyrata*	5.03/25.370	6.22/33.633	119	12.58	EVETLPEEAFEEEEDK
								EILENHGGEER
								IMDEAVNASR
**9↑**	gi/ 297824991	Predicted protein	*Arabidopsis lyrata*	5.26/25.967	7.97/39.819	58	5.54	QVVDETEPK
								VYGSIEEHYYR
**10↓**	gi/2970051	ARG 10	*Vigna radiata*	5.33/30.326	5.62/25.480	133	14.34	DEIFCLFEGALDNLGSLR
								VVCHLSGSFAFIVFDK
**12↑**	gi/416640	Auxin induced protein	*Vigna radiata*	4.81/19.354	4.65/21.345	104	11.34	EGLGLEITELR
								GYSDLAFALEK

* Arrows indicate up- (↑) and down-represented (↓) proteins. Functional categories are according to Bevan *et al.* [18].

### 3.2. RT-PCR Analyses of Cowpea Root Gene Expression

**Figure 3:**
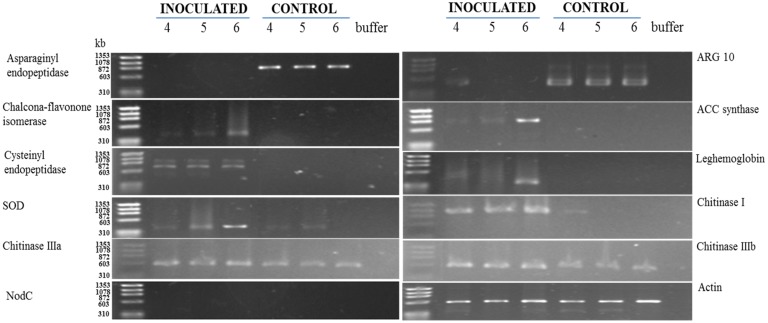
Kinetics of gene expression in the roots of the cowpea genotype CE-31 inoculated with *M. incognita* (Race 3) and the non-inoculated control. Roots were examined at the times indicated (4, 5 and 6 DAI) at the top of the figure. For experimental details, see the Experimental Section.

To validate the results obtained from the proteomic analysis, the levels of asparaginyl endopeptidase, ACC synthase, cysteinyl endopeptidase, chalcone-flavonone isomerase, ARG 10, ascorbate peroxidase (APX), superoxide dismutase (SOD) and leghemoglobin were analyzed by RT-PCR (Figure 3).

The transcript levels of asparaginyl endopeptidase (Spot 1) and ARG 10 (Spot 10) were downregulated between 4 and 6 days after *M. incognita* inoculation (Figure 3), as yet observed by 2D electrophoresis (Figure 2 and Figure 3). On the other hand, increased accumulation patterns both at the protein (Figure 2: two-fold or more up-represented) and/or transcript levels (Figure 3) were observed for ACC synthase (Spot 2), cysteinyl endopeptidase (Spot 4), chalcone-flavone isomerase (Spot 6), Cu,Zn*-*superoxide dismutase (Spot 15), leghemoglobin (Spot 21) and chitinase type I, type IIIa and type IIIb. Amplification of *actin* cDNA, used as the endogenous control [23], showed homogeneity in RKN-inoculated and mock-inoculated cowpea root samples.

## 4. Discussion

In this study, 32 root proteins from the resistant cowpea cv. CE-31 were differentially regulated after challenge with the nematode *M. incognita* (Race 3). This genetic reprogramming was more noticeable between the fourth and sixth day after inoculation (DAI). Using a soybean genome array, Das *et al.* [7] showed that at 9 DAI, 141 genes were 1.5-fold or more upregulated, whereas 59 genes were downregulated in the *M. incognita* inoculated compared with the non-inoculated root of the resistant cowpea access CB46. These differences compared with our results might be due to the techniques used, the period examined, cowpea access-specific defense responses and, also, because we took into consideration protein spots that were two-fold or more up- or down-regulated after RKN inoculation compared with the corresponding control (non-inoculated). Certainly, using other quantitative mass spectrometry methods, such as multiplexed in-gel proteomics, label-free and selective or nonselective labeling of proteins, a much greater number of differential expressed proteins could have been identified in our study [24].

The asparaginyl endopeptidase level decreased within this time interval, compared with the non-inoculated controls (Spot 1: Figure 1; Figure 2; Table 3). It has been experimentally suggested that enzymes of this family also catalyze the transpeptidation by forming a peptide bond, leading to cyclization, as in the case of cyclotides [25]. Cyclotides belongs to a large family of macrocyclic plant proteins of 28–37 amino acids, with three intramolecular disulfide bonds. They have hemolytic, cytotoxic, antimicrobial, insecticidal and have molluscicidal and nematocidal activities, and in plants, their presumed role is to act as antibiotic agents to protect plants from pests or pathogens [26]. Taking into consideration the effects of cyclotides, the downregulation of asparaginyl endopeptidase observed in the cowpea roots challenged with *M. incognita* seems contradictory. Although some caution is needed while interpreting these results, the suppression of asparaginyl endopeptidase expression in the cowpea genotype CE-31 roots could be a nematode strategy to avoid damage by cyclotides. As a parasite, nematodes must protect themselves against plant defenses. Indeed, the potential ability of nematodes to mimic signals in natural plant pathways that manipulate various aspects of plant physiology, including plant defense responses, has been suggested [4]. A study of the *M. incognita* secretome by mass spectrometry identified 486 proteins, and several of these secreted proteins were homologous to plant proteins, which they may mimic, and contain domains that suggest effector functions toward regulating the plant cell cycle or growth, while others have regulatory domains that could reprogram host cells for its own purposes [27].

On the other hand, cysteinyl endopeptidase (Spot 4: Figure 1; Figure 2; Table 3), another proteinase, showed a strong accumulation in the roots of cowpea genotype CE-31 inoculated with *M. incognita*. In regard to the action of cysteine proteinases on nematodes, there is a patent for which it is proposed to use formulations based on at least one plant cysteine proteinase or active fragments to potentiate the anti-nematode effects of non-enzymatic nematicides [28]. Accordingly, cysteine proteinases from papaya latex, papain, stem bromelain and kiwi fruits could effectively reduce nematode infestation of host plants, as the cysteine proteinase attacks the protective cuticle of the nematode, causing blistering, and, eventually, total digestion. Therefore, the increased expression of cysteinyl endopeptidase demonstrated in this present work may have bearing on the resistance of the cowpea genotype CE-31 to *M. incognita* (Race 3).

ACC synthase (Spot 2: Figure 1; Figure 2; Figure 3; Table 3) and ACC oxidase (Spot 3: Figure 1; Figure 2; Table 3) were significantly upregulated, as observed by 2D gels of cowpea roots inoculated with root-knot nematodes compared to controls. The increased level of ACC synthase was also verified by RT-PCR, particularly at 6 DAI (Figure 3). ACC synthase is a key enzyme involved in the ethylene biosynthesis in plants. This sequential increase in ACC synthase and ACC oxidase suggests that the ethylene biosynthetic route was activated upon infection of the resistant cowpea CE-31 with *M. incognita* (Race 3). Thus, the increase in ethylene production after inoculation with *M. incognita* might contribute to the resistance of cowpea CE-31 to this nematode species. In contrast, in a compatible interaction, ACC oxidase was downregulated in the giant cells and surrounding cells seven days post-infection of *Medicago truncatula* cv. Jemalong A17, also a leguminous plant, with *M. incognita*, as observed by microarray hybridization using the Affymetrix GeneChip^®^Medicago genome [29]. According to these authors, this localized repression of the plant defense genes in cells of the host plant, *Medicago truncatula*, in direct contact with the nematode is in accordance with an effective suppression of defenses by secreted effectors of the pathogen, as previously commented. Nevertheless, as for other plant species, increased accumulation of defense transcripts of cowpea against *M. incognita* might result from gene regulation also by ethylene, although different plants may utilize different pathways for defense against a pathogen. Glazer *et al.* [30] have previously suggested that ethylene was closely associated with *M. javanica* infection, as infected tomato plants produced ethylene at a higher rate than uninfected plants and contained higher levels of the ethylene precursor, ACC.

There was a decrease in the abundance of the auxin downregulated ARG10 homologue (Spot 10: Figure 1; Figure 2; Table 3). Moreover, an auxin-induced protein (Spot 12: Figure 1; Figure 2; Table 3) was also upregulated upon *M. incognita* infection of cowpea CE-31. These findings suggest that the auxin level was augmented upon RKN-infection. The establishment and maintenance of nematode feeding sites are strongly influenced by the host plant ethylene and auxin signal transduction pathways [31].

In our previous studies with the pathosystem cowpea genotype CE-31 x *M. incognita*, the activities of the anti-oxidative enzymes, guaiacol peroxidase (POX) and superoxide dismutase (SOD), and those of the PR-proteins, β-1,3-glucanase (GLU), chitinase (CHI) and the cysteine protease inhibitor, were induced in the roots, within 4–8 DAI [10]. Using the proteomic approach and/or RT-PCR, it was confirmed here that CuZnSOD (Spot 15: Figure 1; Figure 2; Figure 3; Table 3), CHI (Spot 18: Figure 1; Figure 2; Figure 3; Table 3) and GLU (Spot 19: Figure 1; Figure 2; Table 3) were upregulated from 4 to 6 DAI. In addition, ascorbate peroxidase (Spot 11: Figure 1; Figure 2; Table 3) was also upregulated in RKN-inoculated cowpea cv. CE-31 in comparison with uninoculated controls. Copper/zinc superoxide dismutase (CuZnSOD) and APX are involved, together with other enzymes, such as CAT, glutathione peroxidase (GPX) and peroxiredoxin (PrxR), in the reactive oxygen species (ROS) network, more precisely with the fine control of hydrogen peroxide (H_2_O_2_) generation in plants, as SOD catalyzes the dismutation of superoxide anions to H_2_O_2_ and O_2_, while APX converts H_2_O_2_ to water. H_2_O_2_ is a second messenger central in the activation of the mitogen-activated protein kinase (MAPK) cascade in plants. H_2_O_2_ is also involved in the cross-linking of cell wall proteins and plant cell wall bound-phenolics, lipid peroxidation, DNA and protein damage, HR, PCD and activation of defense genes and has microbicidal functions [32]. Accumulation of H_2_O_2_ in the leaves of the highly resistant (CE-31) cowpea genotype inoculated with *M. incognita* was previously noticed between 4 and 6 DAI and its decrease between 6 and 8 DAI [10]. *M. incognita* is a biotrophic organism, and therefore, tissue necrosis at the attempted site of nematode fixation caused by ROS during pathogen infection might increases host resistance. However, the persistence of high H_2_O_2_ levels could lead to excessive necrosis of the plant tissue. Thus, it is possible that at this stage (4–8 DAI), APX was enhanced in the studied cowpea to control excessive H_2_O_2_ generated by SOD activity and avoid excessive damage of the plant tissue. Indeed, in our previous enzyme kinetic studies, persistent high levels of SOD activity in the cowpea CE-31 roots between 2 and 10 DAI with *M. incognita* were also shown [10]. In soybean (*Glycine max*) roots infected with *M. incognita*, the increased SOD activity of the resistant centennial cultivar was also observed within 2–7 DAI over that of the respective uninoculated control [33].

The proteomic study of the cowpea CE-31 roots infected with *M. incognita* showed that chalcone-flavonone isomerase (CFI) significantly increased (Spot 6: Figure 1; Figure 2; Table 3) in relation to that of control plants. This finding at the protein level was in agreement with the gene induction observed by RT-PCR (Figure 3). CFI is directly related to the phenylpropanoid biosynthetic pathways, as it accelerates the spontaneous additional cyclization of chalcones to form the flavonoid core from which the antimicrobial compounds, phytoanticipins (constitutive) and phytoalexins (infection-induced), besides tannins and lignin, which also take part in the defense arsenal of plants, are derived [34]. Isoflavone reductase is a key enzyme involved in phytoalexin biosynthesis [34].

Increased accumulation of PR-1 (Spot 17: Figure 1; Figure 2; Table 3) and PR-2 (β-1,3-glucanase) (Spot 19: Figure 1; Figure 2; Table 3) in the roots of RKN-inoculated cowpea CE-31 was also noticed in comparison with control plants. Similarly, three PR-3 (chitinase) isoforms (class I, IIIa and IIIb) were upregulated, particularly chitinase I, as shown both by 2D electrophoresis (Spot 18: Figure 1; Figure 2; Table 3) and RT-PCR (Figure 3). In soybean challenged with *Meloidogyne incognita*, three chitinase isozymes with isoelectric points (pIs) of 4.8, 4.4 and 4.2 accumulated to a greater extent in the resistant (cv. Bryan) compared to the susceptible (cv. Brim) cultivar [35]. This increased accumulation of β-1,3-glucanase (PR-2) and chitinases (Figure 2) in cowpeas is in agreement with the time-course increase previously observed in the cowpea CE-31 roots by our research group [10]. PR-1, PR-2 and PR-3 belong to a protein group, designated pathogenesis-related proteins (PR-proteins), first discovered as being induced in tobacco mosaic virus (TMV)-infected tobacco plants and originally classified into five main groups (PR-1 to PR-5), based on decreasing electrophoretic mobility, but that today encompass seventeen classes numbered in the order of their discovery from PR-1 to PR-17 [36]. The PR-1 family is a dominant, highly conserved group of PRs in plants, induced by pathogens or salicylic acid (SA) and often associated with the establishment of systemic acquired resistance (SAR). Thus, it is plausible to speculate that the upregulation of PR-1 in the cowpea CE-31 root infected with *M. incognita* is associated with systemic acquried resistance (SAR).

Chitinases (PR-3) are enzymes that hydrolyze the beta-1,4-glycosidic linkage of chitin, present in filamentous fungi, insects and nematode eggshells. A great variety of studies have shown that chitinases play an important role in plant defense against biotic stresses. Of particular interest is that the development of eggs and hatching of *M. javanica* juveniles was blocked by proteases and chitinases secreted by *Paecilomyces lilacinus*, a parasite fungus that infects and assimilates eggs of the nematodes, *Meloidogyne* spp., *Globodera* spp. and *Heterodera* spp., as these enzymes drastically altered the eggshell structures when applied individually or in combination [37]. Therefore, overrepresentation of chitinase in the resistant cowpea cv. CE-31 might interfere with the morphofunctional state and hatching of nematode eggs.

β-1,3-Glucanases hydrolyzes β-1,3-glucans and represent the family of PR-2 proteins. Overaccumulation of β-1,3-glucanases together with upregulation of chitinases (PR-3) in response to various pathogen and insect attack has been reported to occur in several plants. PR-2 and PR-3 might contribute to plant defense by acting directly on the pathogen structure, leading to the release of elicitors, or eventually to pathogen death, or they can degrade endogenous plant substrates to generate signal molecules that may function as endogenous elicitors of active host defensive mechanisms [36].

In this present study, a nucleotide-diphosphate kinase (NDPK) was also overexpressed in the roots of the cowpea cv. CE-31 inoculated with *M. incognita*, when compared with mock-inoculated plants (Spot 22: Figure 1; Figure 2; Table 3). NDPKs catalyze the exchange of phosphate groups between different nucleoside diphosphates. Three groups of NDPKs (NDPK1, NDPK2, NDPK3) exist in plants. NDPK1 is localized in the cytosol, NDPK2 in the chloroplast stroma and NDPK3 in the chloroplasts (low abundance) and mitochondria (high abundance) [38]. As more than half of the NDPK transcript pool is represented by the cytosolic NDPK1 in the inflorescence, leaves and roots of *Arabidopsis thaliana* [37], it is supposed that the NADPK overexpressed in the cowpea CE-31 root challenge with *M. incognita* represents the NDPK1 group. Nevertheless, plant NDPKs have been implicated in signal transduction events, UVB light signaling, hormone, heat shock response and interaction and seem to be involved in the mitogen-activated protein kinase (MAPK) pathway signaling [38]. TAB2, an NDPK of tomato, upregulated the expression of PR-1, PR-2 (β-1,3-glucanases) and PR-3 (chitinases) genes. Interestingly, a human NDPK isoform (Nm23) is a strong metastatic tumor suppressor [39]. In a compatible reaction of *M. incognita* with a host plant, one of the characteristic symptoms observed in the infected roots is the formation of the typical root gall (tumors) resulting from hyperplasia and hypertrophy of the cells surrounding the nematode feeding sites (giant cells) [1]. In the CE-31 resistant cowpea genotype, gall formation was a rare event [10].

A 17.5-kDa heat shock protein (HSP) class I (CI) was also upregulated in the cowpea CE-31 roots infected with *M. incognita* (Spot 16: Figure 1; Figure 2; Table 3). Based on its molecular mass, this HSP could be classed as small heat shock proteins (sHSPs). sHSPs are numerous and very diverse, both in sequence and where they function in the cell [40]. HSPs belong to a well-conserved class of molecules that function as molecular chaperones, playing key roles in protein folding and refolding, assembly and transport, stabilization of proteins and membranes under stress conditions and in the reestablishment of cellular homeostasis. Additionally, it has been reported that biotic stress can induce the gene expression of some, but not all, sHSPs [40].

Surprisingly, in the cowpea cv. CE-31 roots challenged with *M. incognita*, but not in control plants, there was the induction of the leghemoglobin (LegHb) biosynthesis (Spot 21: Figure 1; Figure 2; Table 3), which was linked to gene activation (Figure 3). To the best of our knowledge, this is the first time that induction of a LegHb by RKN-infection of non-rhizobium bacterized cowpea has been reported. Since the cowpea plants were grown in autoclaved sand, the root system of the studied cowpea was not rhizobium bacterized, as proven by the absence of amplification (transcripts) of the NodC genes assessed by PCR using specific nucleotide primers from conserved regions of the *nodC* gene (Figure 3). Therefore, such upregulation of LegHb in the cowpea CE-31 was due to the nematode infection itself. LegHbs are essential for the symbiotic nitrogen fixation process in the legume root nodules induced by rhizobia, where the main function is to act as a carrier of oxygen from the atmosphere to the bacteroids for aerobic respiration [41]. It was previously shown [42] that cowpea seeds bacterized with a rhizobium strain and inoculated with *M. incognita* had a decrease in the LegHb content over that of the rhizobium bacterized cowpea not RKN-inoculated (control). Kinetic studies have shown that soybean LegHbs decompose H_2_O_2_ to H_2_O with kinetics similar to that for the reactions of plant peroxidases [43]. Thus, it is possible that this enhanced legHb detected in our study must be also involved in the H_2_O_2_ homeostasis of cowpea plants infected with *M. incognita*. Nevertheless, further studies are needed to clarify both why LegHb was induced upon *M. incognita* challenge in non-rhizobium bacterized cowpea and what real physiological function LegHb plays within this scenario.

## 5. Conclusions

**Figure 4:**
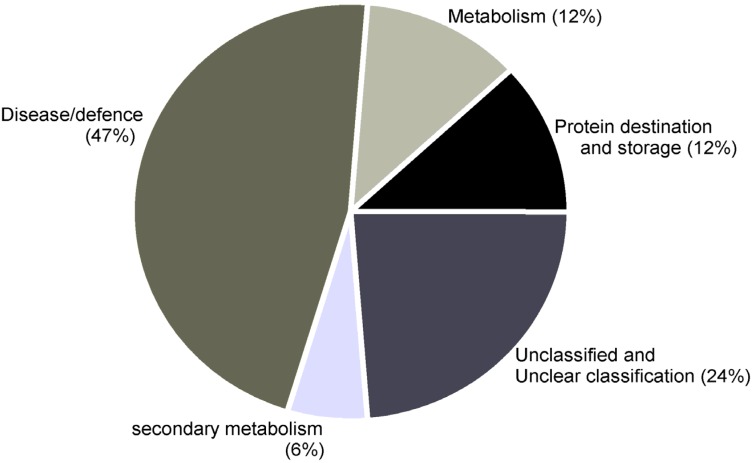
Gene ontology (GO) for proteins in the roots of cowpea genotype CE-31 inoculated with *M. incognita* (Race 3) and non-inoculated control. Categories are according to [18] and represented as a percentage of total identified GO terms.

In conclusion, this work shows that the defense response of the resistant cowpea CE-31 to infection by root-knot nematodes, *M. incognita*, is complex and involves many different proteins and metabolic pathways (Figure 4). Nevertheless, the upregulated proteins, such as SOD, APX, PR-1, β-1,3-glucanase, chitinases, cysteine protease and secondary metabolism enzymes, key enzymes involved in the ethylene biosynthesis in plants, and proteins involved in the MAPK pathway signaling, amongst others, reinforce that they may contribute, directly or indirectly, to the resistance of cowpea to *M. incognita* attack.

## References

[1] Castagnone-Sereno P., Danchin E.G.J., Perfus-Barbeoch L., Abad P. (2013). Diversity and evolution of root-knot nematodes, genus *Meloidogyne*: New insights from the genomic era. Annu. Rev. Phytopathol..

[2] Schulze-Lefert P., Ralph P. (2011). A molecular evolutionary concept connecting nonhost resistance, pathogen host range, and pathogen speciation. Trends Plant Sci..

[3] Quentin M., Abad P., Favery B. (2013). Plant parasitic nematode effectors target host defense and nuclear functions to establish feeding cells. Front. Plant Sci..

[4] Gheysen G., Mitchum M.G. (2011). How nematodes manipulate plant development pathways for infection. Curr. Opin. Plant Biol..

[5] Heil M., Bostock R.M. (2002). Induced systemic resistance (ISR) against pathogens in the context of induced plant defences. Ann. Bot..

[6] Barna B., Fodor J., Harrach B.D., Pogány M., Király Z. (2012). The Janus face of reactive oxygen species in resistance and susceptibility of plants to necrotrophic and biotrophic pathogens. Plant Physiol. Biochem..

[7] Das S., Ehlers J.D., Close T.J., Roberts P.A. (2010). Transcriptional profiling of root-knot nematode induced feeding sites in cowpea (*Vigna unguiculata* L. Walp.) using a soybean genome array. BMC Genomics.

[8] Souza e Silva S.M., Maia J.M., Araújo Z.B., Freire-Filho F.R. (2002). Chemical Composition of 45 Cowpea [Vigna unguiculata (L.) Walp.] Genotypes.

[9] Ehlers J.D., Matthews W.C., Hall A.E., Roberts P.A., Fatokun C.A., Tarawali S.A., Singh B.B., Kormawa P.M., Tamo M. (2002). Breeding and evaluation of cowpeas with high levels of broad-based resistance to root-knot nematodes. Challenges and Opportunities for Enhancing Sustainable Cowpea Production, Proceedings of the World Cowpea Conference III.

[10] Oliveira J.T.A., Andrade N.C., Martins-Miranda A.S., Soares A.A., Gondim D.M.F., Araújo-Filho J.H., Freire-Filho F.R., Vasconcelos I.M. (2012). Differential expression of antioxidant enzymes and PR-proteins in compatible and incompatible interactions of cowpea (*Vigna unguiculata*) and the root-knot nematode *Meloidogyne incognita*. Plant Physiol. Biochem..

[11] Silveira J.A.G., Costa R.C.L., Oliveira J.T.A. (2001). Drought-induced effects and recovery of nitrate assimilation and nodule activity in cowpea plants inoculated with *Bradyrhizobium* spp. under moderate nitrate level. Braz. J. Microbiol..

[12] Hussey R.S., Davis E.L., Baum T.J. (2002). Secrets in secretions: Genes that control nematode parasitism of plants. Braz. J. Plant Physiol..

[13] Atkinson H.J., Gurr S.J., McPherson M.J., Bowles D.J. (1992). Nematodes. Molecular Plant Pathology.

[14] Bradford M.M. (1976). A rapid and sensitive method for the quantification of microgram quantities of protein utilizing the principle of protein dye binding. Anal. Biochem..

[15] Laemmli U.K. (1970). Cleavage of structural proteins during the assembly of the head of bacteriophage T4. Nature.

[16] Candiano G., Bruschi M., Musante L., Santucci L., Ghiggeri G.M., Carnemolla B., Orecchia P., Zardi L., Righetti P.G. (2004). Blue silver: A very sensitive colloidal Coomassie G-250 staining for proteome analysis. Electrophoresis.

[17] Shevchenko A., Wilm M., Vorm O., Mann M. (1996). Mass spectrometric sequencing of proteins from silver-stained polyacrylamide gels. Anal. Chem..

[18] Bevan M., Bancroft I., Bent E., Love K., Goodman H., Dean C., Bergkamp R., Dirkse W., van Staveren M., Stiekema W. (1998). Analysis of 1.9 Mb contiguous sequence from chromosome 4 of *Arabidopsis thaliana*. Nature.

[19] Chang S., Puryear J., Cairney J. (1993). A simple and efficient method for isolating RNA from pine trees. Plant Mol. Biol. Rep..

[20] Sambrook J., Fritsch E.F., Maniatis T. (1989). Molecular Cloning: A Laboratory Manual.

[21] Sarita S., Sharma P.K., Priefer U.B., Prell J. (2005). Direct amplification of rhizobial *nodC* sequences from soil total DNA and comparison to *nodC* diversity of root nodule isolates. FEMS Microbiol. Ecol..

[22] Warner S., Foster G.D., Twell D. (1996). Genomic DNA isolation and lambda library construction. Plant Gene Isolation: Principles and Practice.

[23] Costa J.H., Hasenfratz-Sauder M.P., Pham-Thi A.T., Silva Lima M.G., Dizengremel P., Jolivet Y., Fernandes de Melo D. (2004). Identification in *Vigna unguiculata* (L.) Walp. of two cDNAs encoding mitochondrial alternative oxidase orthologous to soybean alternative oxidase genes 2a and 2b. Plant Sci..

[24] Twyman R.M. (2014). Strategies for protein quantitation. Principles of Proteomics.

[25] Xu W., Li L., Du L., Tan N. (2011). Various mechanisms in cyclopeptide production from precursors synthesized independently of non-ribosomal peptide synthetases. Acta Biochem. Biophys. Sin..

[26] Craik D.J. (2012). Host-defense activities of cyclotides. Toxins.

[27] Bellafiore S., Shen Z., Rosso M.-N., Abad P., Shih P., Briggs S.P. (2008). Direct identification of the meloidogyne incognita secretome reveals proteins with host cell reprogramming potential. PLoS Pathog..

[28] Curtis R., Buttle D., Behnke J., Duce I., Shewry P., Kurup S., Kerry B., Kerry M. (2008). Nematicidal effects of cysteine proteinases and methods of use thereof to treat nematode infestation.

[29] Damiani I., Baldacci-Cresp F., Hopkins J., Andrio E., Balzergue S., Lecomte P., Puppo A., Abad P., Favery B., Hérouart D. (2012). Plant genes involved in harbouring symbiotic rhizobia or pathogenic nematodes. New Phytol..

[30] Glazer I., Epstein E., Orion D., Apelbaum A. (1986). Interactions between auxin and ethylene in root-knot nematode (*Meloidogyne javanica*) infected tomato roots. Physiol. Mol. Plant Pathol..

[31] Gutierrez O.A., Wubben M.J., Howard M., Roberts B., Hanlon E., Wilkinson J.R. (2009). The role of phytohormones ethylene and auxin in plant-nematode interactions. Russ. J. Plant Physiol..

[32] Neil S.J., Desikan R., Clarke A., Hurst R.D., Hancock J.T. (2002). Hydrogen peroxide and nitric oxide as signaling molecules in plants. J. Exp. Bot..

[33] Vanderspool M.C., Kaplan D.T., McCollum T.G., Wodzinski R.J. (1994). Partial characterization of cytosolic superoxide dismutase activity in the interaction of *Meloidogyne incognita* with two cultivars of *Glycine max*. J. Nematol..

[34] Ferrer J.-L., Austin M.B., Stewart C., Noel J.P. (2008). Structure and function of enzymes involved in the biosynthesis of phenylpropanoids. Plant Physiol. Biochem..

[35] Qiu J., Hallmann J., Kokalis-Burelle N., Waever D.B., Rodríguez-Kábana R., Tuzun S. (1997). Activity and differential induction of chitinase isozymes in soybean cultivars resistant or susceptible to root-knot nematodes. J. Nematol..

[36] Van Loon L.C., Rep M., Pieterse C.M. (2006). Significance of inducible defense-related proteins in infected plants. Annu. Rev. Phytopathol..

[37] Khan A., Williams K.L., Nevalainen H.K.M. (2004). Effects of *Paecilomyces lilacinus* protease and chitinase on the eggshell structures and hatching of *Meloidogyne javanica* juveniles. Biol. Control.

[38] Hammargren J., Sundström J., Johansson M., Bergman P., Knorpp C. (2007). On the phylogeny, expression and targeting of plant nucleoside diphosphate kinases. Physiol. Plantarum.

[39] Steeg P.S., Bevilacqua G., Kopper L., Thorgeirsson U.P., Talmadge J.E., Liotta L.A., Sobel M.E. (1988). Evidence for a novel gene associated with low tumor metastatic potential. J. Natl. Cancer Inst..

[40] Waters E.R. (2013). The evolution, function, structure, and expression of the plant sHSPs. J. Exp. Bot..

[41] Ott T., van Dongen J.T., Gunther C., Krusell L., Desbrosses G., Vigeolas H., Bock V., Czechowski T., Geigenberger P., Udvarvi M.K. (2005). Symbiotic leghemoglobins are crucial for nitrogen fixation in legume root nodules but not for general plant growth and development. Curr. Biol..

[42] Khan T.A., Husain S.I. (1989). Proline and leghaemoglobin contents and water absorption capability of cowpea roots as influenced by infection with *Rotylenchus reniformis*, *Meloidogyne incognita* and *Rhizoctonia solani*. Nematol. Medit..

[43] Job D., Zeba B., Puppo A., Rigaud J. (1980). Kinetic studies of the reaction of ferric soybean leghemoglobins with hydrogen peroxide, cyanide and nicotinic acid. Eur. J. Biochem..

